# Predictive underestimation of difficult direct laryngoscopy in a patient with rheumatoid arthritis-associated immobilized craniocervical junction

**DOI:** 10.1186/s40981-023-00679-9

**Published:** 2023-12-05

**Authors:** Hirotaka Matsuyama, Masato Hara, Atsushi Seto, Teruyuki Hiraki

**Affiliations:** 1 Department of Anesthesiology, Kurume University School of Medicine, 67 Asahi-Machi, Kurume, Fukuoka 830-0011 Japan; 2 Nagata Orthopedic Hospital, 1-6-3 Shiranui-Machi, Omuta, Fukuoka 836-0843 Japan

**Keywords:** Rheumatoid arthritis, Difficult intubation, Upper cervical spine mobility, Neck rotation, Airway Scope

## Abstract

**Background:**

The upper cervical spine is a major focus of damage by rheumatoid arthritis (RA). Specific screening for mobility of the upper cervical spine, which is essential for direct laryngoscopy, is lacking. Herein, we present a case of RA with Cormack-Lehane grade IV, which was not predicted by preoperative examination.

**Case presentation:**

A 66-year-old woman with RA was scheduled for a right total knee arthroplasty and right elbow synovectomy. She had a long history of RA without symptoms related to the cervical spine or spinal cord. Although physical examination suggested moderate risk of difficult intubation with preserved cervical retroflexion, her Cormack-Lehane classification was grade IV under muscle relaxation. Bony integration of the occiput to axis was considered to be the main cause of difficult direct laryngoscopy, and restricted neck rotation was found postoperatively.

**Conclusions:**

RA patients may have limited upper cervical spine motion despite normal cervical retroflexion.

## Background

Predicting difficult airway is an important role of preoperative examination. The upper cervical spine is a major focus of damage by rheumatoid arthritis (RA). However, a technique for specific screening for mobility of the upper cervical spine, which is essential for direct laryngoscopy, is lacking. In this report, we describe a case of RA with Cormack-Lehane grade IV (epiglottis is not visible), which was not predicted by preoperative examination.

## Case presentation

A 66-year-old woman (height 159 cm, weight 45 kg) suffering from right elbow and right knee deformity associated with RA was scheduled for a right total knee arthroplasty and right elbow synovectomy. She had a long history of RA and had been aware of joint pain in her right elbow and right knee for 30 years. She had been receiving medical treatment for 15 years prior to the surgery. She did not report any symptoms related to the cervical spine or spinal cord.

**Fig. 1:**
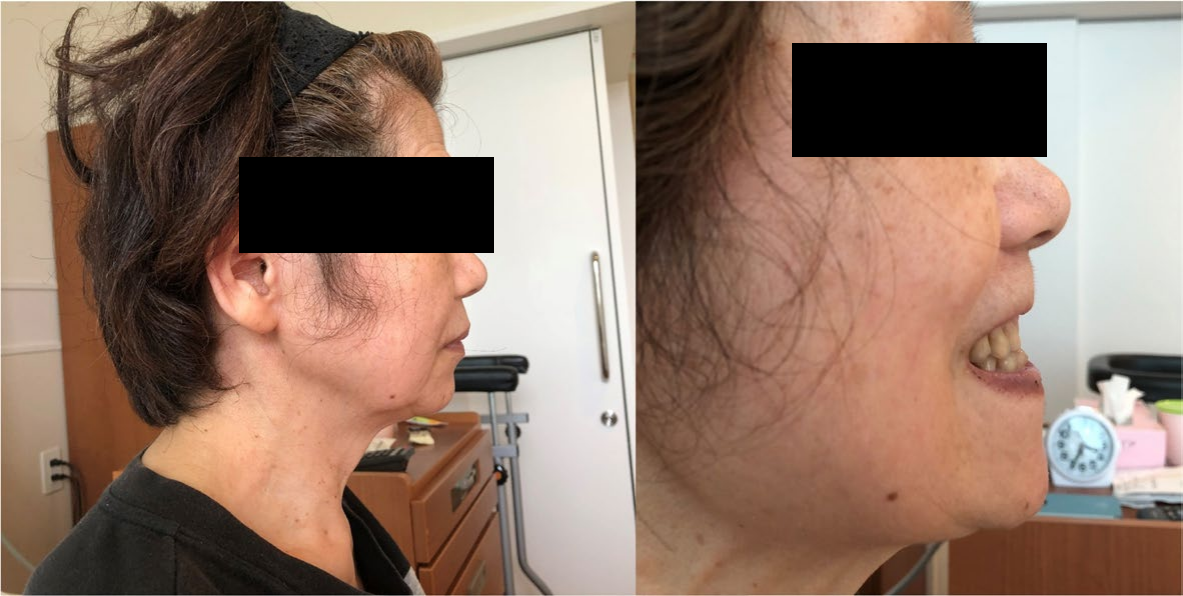
Patient at lateral neutral position (left) and at mandibular protrusion test (right). No obvious micrognathia, retrognathia, or short neck is found. She is equivalent to mandibular protrusion test class B, with moderate limitation of forward movement

On preoperative examination, the patient appeared to have normal mandibular size, without retrognathia, and adequate thyromental distance, despite her overall small stature and small facial features (Fig. 1). The opening was two transverse fingers. Mallampati classification was class III, with a slightly visible base of the palatine roof at opening. Cervical retroflexion was judged to be unrestricted. Based on these findings, we predicted moderate intubation difficulty in this patient.

General anesthesia with tracheal intubation and echo-guided peripheral nerve blocks for axillary brachial plexus and femoral nerve were planned. In the operating room, standard monitoring was applied followed by administration of the echo-guided peripheral nerve blocks. General anesthesia was induced with propofol 90 mg and rocuronium 50 mg, and after confirmation of mask ventilation, continuous remifentanil 0.25 µg/kg/min was started. Direct visualization of the larynx with the direct laryngoscope was more difficult than predicted; the epiglottis was not visible with thyroid cartilage compression, indicating Cormack-Lehane grade IV. Using a video laryngoscope Airway Scope® AWS-S200 (Pentax, Tokyo, Japan), the glottis was fully visualized, and the trachea was easily intubated. After the surgery, the patient was extubated uneventfully and had a safe postoperative period.

**Fig. 2:**
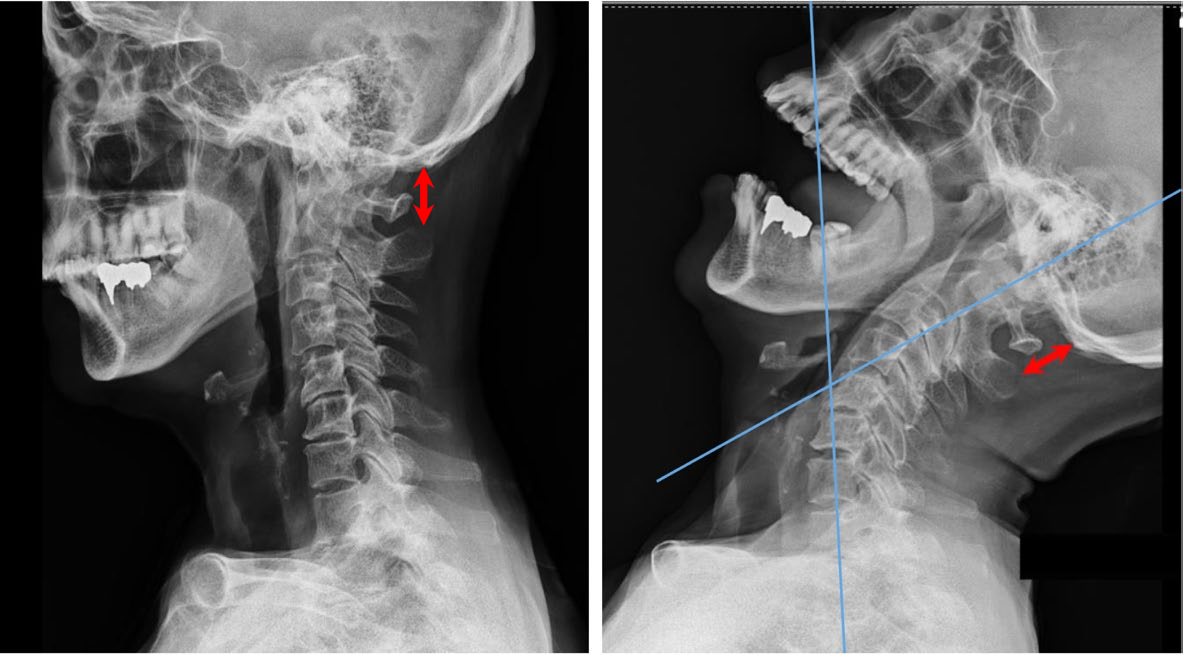
Plain radiograph (lateral view) in neutral position (left) and maximum neck retroflexion (right). The distance between the occiput and the spinous process of the axis is not shortened by maximum cervical retroflexion (bidirectional red arrows, same size on both sides). Blue lines represent the axis of the lower spine and the axis of the upper spine. Maximum retroflexion of the entire neck is 64° (right)

**Fig. 3:**
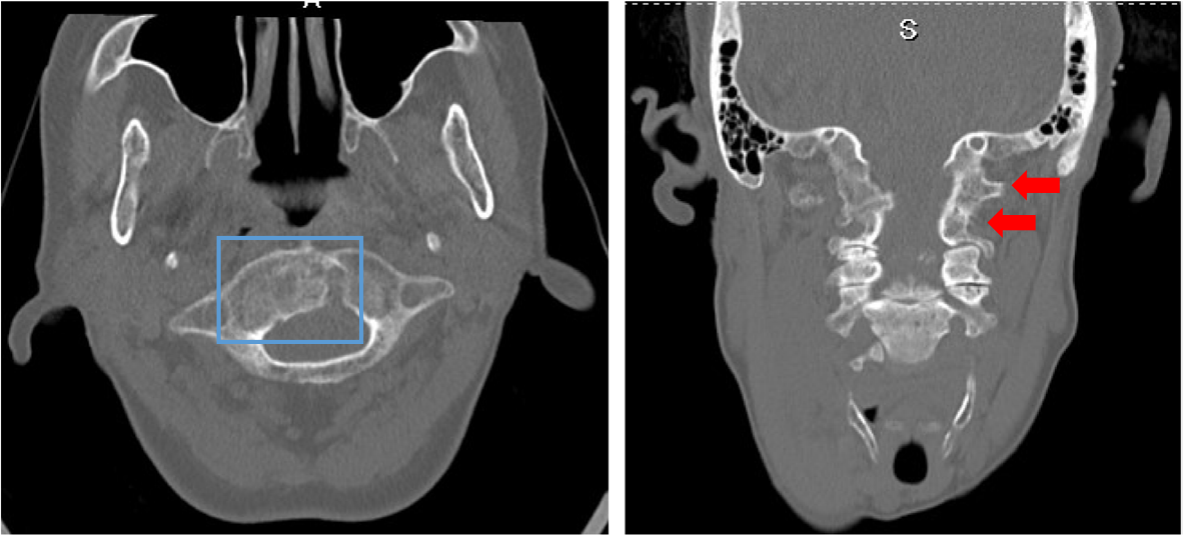
Computed tomography of the head and cervical spine in an axial slice at the atlas vertebra (left) and a coronal slice at the spinal canal (right). The blue box indicates a bony integration of the odontoid process of the axis and the atlas. The red arrows indicate the vertebral arches of the atlas and axis, respectively. The boundary between the occiput, atlas and axis is indistinct, suggesting bony integration

We performed postoperative radiographic examination for elucidating the causes of difficult laryngoscopy with the consent of the patient. Cervical radiograph demonstrated no change in the distance between the occiput and the spinous process of the axis with neck retroflexion (Fig. 2), suggesting an immobilized craniocervical junction. Computed tomography revealed a bony integration of the occiput, atlas, and axis, which may have limited the range of motion of the upper cervical spine and was thought to be the primary cause of difficult direct laryngoscopy (Fig. 3).

**Fig. 4:**
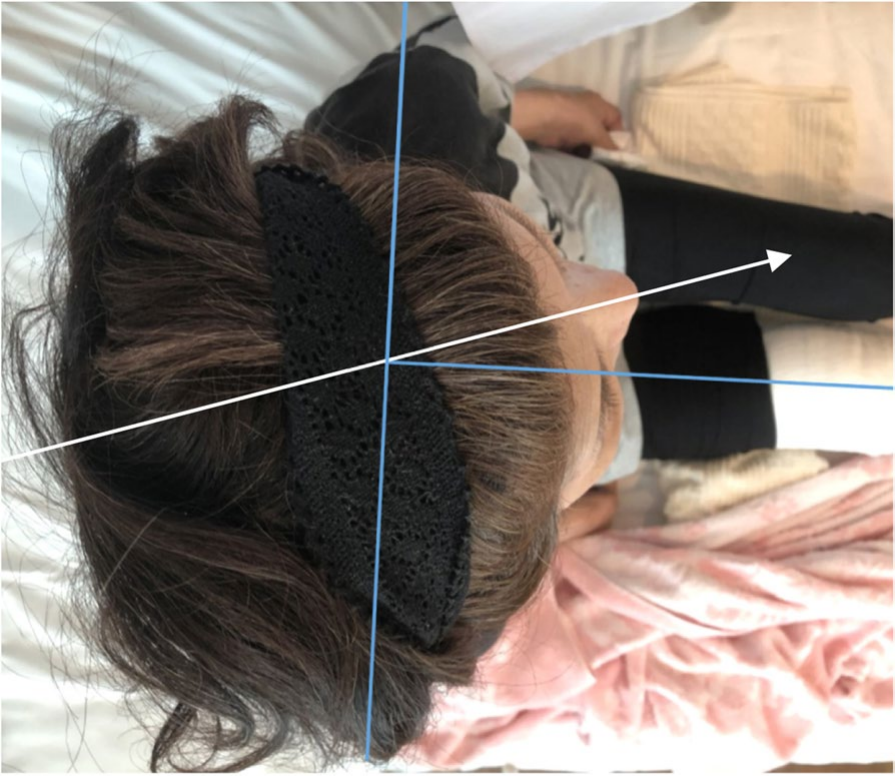
Overhead view of maximum cervical rotation to the left. The blue lines indicate the sagittal and the coronal lines of the patient’s body. The arrow indicates the sagittal line of her head. Maximum rotation of the neck is 18°

In order to verify whether the difficulty in direct laryngoscopy was predictable, another postoperative physical examination was performed with the patient’s consent. The mandibular protrusion test was class B, with the mandibular incisors in contact with the maxillary incisors [1] (Fig. 1). Cervical retroflexion (64°) was obtained (Fig. 2); however, cervical rotation was highly restricted (18°) (Fig. 4). The thyromental distance was 6.5 cm, the hyomental distance was 4 cm, and the sternomental distance was 14.5 cm, suggesting moderate difficulty of tracheal intubation.

## Discussion

Direct laryngoscopy in this case was more difficult than predicted and was Cormack-Lehane grade IV. Among the preoperative findings, Mallampati class III was predictive of difficult intubation. However, it has been noted that a single bedside screening test is not sufficient to predict difficult intubation [2]. The sensitivity of Mallampati class III or higher for predicting difficult intubation derived from 47 studies involving 23,396 patients was reported to be 0.55 [3]. Therefore, several physical findings are combined in common clinical practice. In the “JSA airway management guideline 2014” [4], the Japanese Society of Anesthesiologists proposed 12 preoperative assessment factors that predict combined difficult facemask ventilation and direct laryngoscopy. In the present case, four of the 12 items were consistent: Mallampati class III, age over 46 years, presence of teeth, and limitation of forward mandibular movement (mandibular protrusion test class B). The likelihood of simultaneous occurrence of difficult mask ventilation and difficult direct laryngoscopy in this model was 0.47%, indicating that it was difficult to predict based on physical findings alone. A recent systematic review listed inability of upper lip bite, a short hyomental distance, and retrognathia as the best predictors of difficult endotracheal intubation [3]. Thus, mandibular size and forward mobility are strongly associated with difficulty in direct laryngoscopy. Although the upper lip bite was untested in this case, the forward mobility limitation of the mandible was not severe.

Ul Haq et al. reported that patients with Mallampati class III and IV have a 6.38-fold increased risk of difficult intubation, and patients with mandibular protrusion test class B and C (lower incisors cannot be protruded edge to edge with upper incisors) have an 8.32-fold increased risk of difficult intubation. Of the 760 patients enrolled in the study, 9.3% had Mallampati class III or IV, and 8.2% had mandibular protrusion test class C; however, none had Cormack-Lehane grade IV [5]. Given that the present patient had a moderate risk of difficult intubation, our findings of Cormack-Lehane grade IV made this an unusual case.

Another significance of this case report is that the present case had difficulty with direct laryngoscopy, probably due to a bone deformity of the upper cervical vertebra, despite normal neck extension. Visualization of the glottis during direct laryngoscopy requires alignment of the operator’s gaze with the glottic axis, which has been shown to be approached by extension of the occipito-atlantal joint in awake healthy volunteers [6]. A study using lateral fluoroscopy to record the movement of the occiput to the cervical spine in patients under general anesthesia also reported that most of the cervical motion during direct laryngoscopy occurs at the occipito-atlantal and atlanto-axial joints [7]. RA is associated with cervical spine involvement in up to 80% of patients, with the upper cervical spine being particularly susceptible [8]. Many anesthesiologists are aware of the possibility of airway compromise due to limited mobility of the cervical spine in patients with RA. However, joint damage is commonly associated with instability, such as subluxation and neurological symptoms. Therefore, the asymptomatic course of this patient may have led to an underestimation of cervical spine deformity. Takenaka et al. reported the limitations of the physical cervical extension test, such as the Bellhouse test, for limited upper cervical spine mobility, and proposed the usefulness of the ratio of hyomental distance in head extension and neutral position (hyomental distance ratio; HMDR) as an airway assessment in RA patients [9]. Although HMDR may be a good predictor of decreased cervical extension capacity, accurate measurement may be difficult to attain. In this case, neck rotation was limited despite preserved cervical retroflexion. The range of motion of neck rotation in adults without cervical spine involvement is approximately 70° to one side [10, 11], so this case is clearly small. Considering that at least 60% of neck rotation is produced by the upper cervical spine [11], a range of motion of 18° in this case may raise suspicion of limited upper cervical motion. Although there is no evidence to support neck rotation as a screening for limited upper cervical motion, it may be useful as a reference to prompt further evaluation by radiography or CT.

Although the patient did not appear to have impaired extension of the entire neck, radiographic examination in the retroflexed position was decisive in diagnosing limited upper cervical motion. The presence of a shortening of the occipito-atlantal or -axial distance in the retroflexed position is directly diagnostic of the extension capacity of the occipito-atlanto-axial complex. CT scans are useful for detailed evaluation of bone and joint deformities.

In conclusion, we present a case of Cormack-Lehane grade IV that was not predicted preoperatively. Anesthesiologists should be aware that RA patients may have limited upper cervical spine motion, which is essential for direct laryngoscopy, despite normal cervical retroflexion. Neck rotation may be useful as a reference to facilitate further assessment of upper cervical spine mobility.

## Data Availability

The data used in the present article are available upon reasonable request.

## Abbreviations

RA: Rheumatoid arthritis
CT: Computed tomography
HMDR: Hyomental distance ratio
